# DXA-Based Detection of Low Muscle Mass Using the Total Body Muscularity Assessment Index (TB-MAXI): A New Index with Cutoff Values from the NHANES 1999–2004

**DOI:** 10.3390/jcm11030603

**Published:** 2022-01-25

**Authors:** Marco Alessandro Minetto, Maria Giulia Ballatore, Alberto Botter, Chiara Busso, Angelo Pietrobelli, Anita Tabacco

**Affiliations:** 1 Division of Physical Medicine and Rehabilitation, Department of Surgical Sciences, University of Turin, 10126 Turin, Italy; chiara.busso@unito.it; 2 Department of Mathematical Sciences, Politecnico di Torino, 10129 Turin, Italy; maria.ballatore@polito.it (M.G.B.); anita.tabacco@polito.it (A.T.); 3 Laboratory for Engineering of the Neuromuscular System (LISiN), Department of Electronic and Telecommunications, Politecnico di Torino, 10129 Turin, Italy; alberto.botter@polito.it; 4 PolitoBIOMed Lab, Politecnico di Torino, 10129 Turin, Italy; 5 Paediatric Unit, Department of Surgical Sciences, Dentistry, Gynaecology and Paediatrics, University of Verona, 37126 Verona, Italy; angelo.pietrobelli@univr.it; 6 Pennington Biomedical Research Centre, Baton Rouge, LA 70808, USA

**Keywords:** appendicular lean mass, dual-energy X-ray absorptiometry (DXA), inter- and intra-muscular adipose tissue (IMAT), National Health and Nutrition Examination Survey (NHANES), sarcopenia

## Abstract

The aims of this study were to investigate age-related changes in total body skeletal muscle mass (TBSMM) and the between-limb asymmetry in lean mass in a large sample of adults. Demographic, anthropometric, and DXA-derived data of National Health and Nutrition Examination Survey participants were considered. The sample included 10,014 participants of two ethnic groups (Caucasians and African Americans). The age-related decline of TBSMM absolute values was between 5% and 6% per decade in males and between 4.5% and 5.0% per decade in females. The adjustment of TBSMM for body surface area (TB-MAXI) showed that muscle mass peaked in the second decade and decreased progressively during the subsequent decades. The following thresholds were identified to distinguish between low and normal TB-MAXI: (i) 10.0 kg/m^2^ and 11.0 kg/m^2^ in Caucasian and African American females; and (ii) 12.5 kg/m^2^ and 14.5 kg/m^2^ in Caucasian and African American males. The lean asymmetry indices were higher for the lower limbs compared with the upper limbs and were higher for males compared with females. In conclusion, the present study proposes the TB-MAXI and lean asymmetry index, which can be used (and included in DXA reports) as clinically relevant markers for muscle amount and lean distribution.

## 1. Introduction

The assessment of lean mass is crucial for the diagnosis of sarcopenia, which consists of the loss of skeletal muscle mass and function that occurs during the aging process (primary sarcopenia) or due to the presence of an underlying disease or medication (secondary sarcopenia) [1,2,3].

Dual-energy X-ray absorptiometry (DXA) is widely adopted in research and clinical settings for the three-compartment model-based assessment of total body lean mass and appendicular lean mass (ALM) [3,4,5,6,7]. The DXA-derived lean indices most commonly included in DXA reports and adopted for the diagnosis of sarcopenia are the absolute values of ALM and the values of ALM normalized to height squared (to account for allometric differences in body size) to obtain the appendicular lean mass index that enables the comparisons among different subjects independently of their body size [4,5,6,7]. However, DXA reports could also be integrated with additional indices that can be useful for diagnosis, prognosis, and follow-up of patients with sarcopenia. 

For example, the asymmetry in lower-limb lean mass showed a significant association with gait speed in older adults [8]. Moreover, high levels of between-limb asymmetry in lower-extremity lean mass were associated with reduced lower-extremity power [9]. The lean mass asymmetry between the two sides can easily be assessed by DXA for both the upper and lower limbs. However, to the best of our knowledge, the between-limb asymmetry index is usually not included in DXA reports, and no normative data exist to distinguish between low (i.e., physiological) and high (i.e., pathological) asymmetry of the lean mass distribution.

Another variable that is commonly not included in DXA reports is the estimate of total body skeletal muscle mass (TBSMM). In this view, Kim et al. developed and validated six different equations to estimate the TBSMM from DXA-derived ALM values [10,11]. To our knowledge, no previous study has been performed to systematically investigate the differences (if any) among the six equations and to assess the age-related decline of TBSMM in a large population of subjects. 

Therefore, the aim of this study was to reduce these gaps by analyzing the between-limb asymmetry in upper- and lower-extremity lean mass and the age-related changes in TBSMM values obtained with different equations in a large sample of subjects of both genders of different ethnic groups.

## 2. Materials and Methods

### 2.1. Study Design

This cross-sectional study used the data obtained from the National Health and Nutrition Examination Survey (NHANES) collected from 1999 to 2004 (accessible online at the following website: https://www.cdc.gov/nchs/nhanes/ accessed on 10 September 2021). 

All participants provided their written informed consent before participating. The 1999–2004 NHANES protocol was approved by the Institutional Review Board of the National Center for Health Statistics, Centers for Disease Control and Prevention (US: protocol #98-12; accessible online at the following website: https://www.cdc.gov/nchs/nhanes/irba98.htm accessed on 28 December 2021).

### 2.2. Participants

In the current study, participants were excluded if they were <18 years, had missing data on our primary variable dataset, or had a body mass index (BMI) <16 kg/m^2^ or >35 kg/m^2^.

Three of the six equations by Kim et al. [10] were developed using a sample of subjects of the following ethnic groups: Caucasian, Hispanic, African American, and Asian. The other three equations by Kim et al. [11] were developed using a sample of subjects of the following ethnic groups: Caucasian, African American, and Asian. Only NHANES participants of the following two ethnic groups were considered in the present study: Caucasian and African American (we excluded Hispanics because three of the six equations were not available for this ethnic group, and we also excluded Asians because adult lean subjects of this ethnic group were under-represented in the 1999–2004 survey cycle). These participants were grouped into the following 7 age categories: <20 years; 20–29 years; 30–39 years; 40–49 years; 50–59 years; 60–69 years; and ≥70 years.

### 2.3. Anthropometry and DXA

Weight, height, and body composition of NHANES participants were evaluated at the Mobile Examination Center using the previously described methodology [12,13] and the following devices: Mettler Toledo digital scale (Mettler Toledo International Inc., Columbus, OH, USA), seca electronic stadiometer (seca GmbH & Co. KG., Hamburg, Germany), and QDR 4500 A fan beam X-ray densitometer (Hologic Inc., Marlborough, MA, USA). 

Anthropometric and body composition data were used to obtain the following variables: total lean mass (TLM) and lean mass index (LMI), appendicular lean mass (ALM) and appendicular lean mass index (ALMI), and lean asymmetry index (AI) for the upper and lower limbs, body surface area (BSA), total body skeletal muscle mass (TBSMM), and total body muscularity assessment index (TB-MAXI).

TLM (kg) was estimated as the sum of the lean mass from the head, trunk, arms, and legs. It was adjusted by height squared to obtain the LMI (kg/m^2^).

ALM (kg) was estimated as the sum of the lean mass from arms and legs. It was adjusted by height squared to obtain the ALMI (kg/m^2^). 

Lean AI (%) was obtained for the upper and lower limbs according to the following equation [8]:

Lean AI = [(Between-limb difference in lean mass)/0.5 ∗ (Sum of the lean mass of the two limbs)] ∗ 100

BSA (m^2^) was estimated according to the following equation [14]:

BSA (m^2^) = 0.007184 ∗ weight (kg)^0.425^ ∗ height (cm)^0.725^ (1)

**Figure 1:**
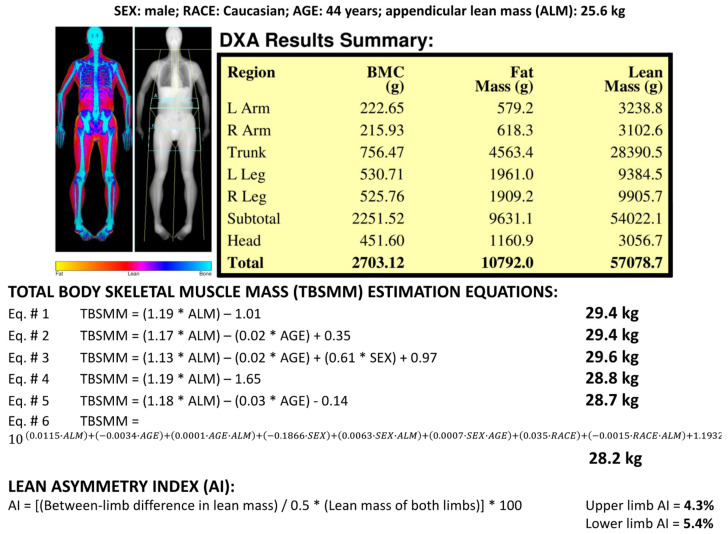
Dual-energy X-ray absorptiometry report of body composition (bone mineral content—BMC; fat mass; lean mass) for one representative Caucasian male subject (age 44 years). Results of the total body skeletal muscle mass (TBSMM) estimation for the representative subject according to the six equations previously proposed by Kim et al. [10,11]. Results of the lean asymmetry index estimation for the upper limbs (4.3%) and for the lower limbs (5.4%) of the representative subject. Coefficients for Equation #3. SEX: female = 0, male = 1. Coefficients for Equation #6. SEX: female = 1, male = 0; RACE: Caucasian = 0, African American = 1.

TBSMM was estimated on the basis of the ALM value and anthropometric variables (i.e., age, gender, and ethnicity) according to the six equations previously proposed by Kim et al. [10,11] (Figure 1). The standard error of estimate for the six equations ranged between 1.06 kg and 1.63 kg [10,11]. TBSMM was adjusted by BSA to obtain the TB-MAXI (kg/m^2^).

### 2.4. Statistical Analysis

Since the Shapiro–Wilk test showed normal distribution of the data, parametric tests were used, and the sample characteristics are reported as means and standard deviations in the tables and are represented as means and 95% confidence intervals in the figures.

A cubic spline interpolation of raw data was adopted to generate ethnic-specific and gender-specific TBSMM curves.

A one-way repeated-measures ANOVA followed by the Holm–Sidak post hoc test was used to check for differences between the TBSMM estimates obtained with the six equations, while a two-way ANOVA was used for comparisons among different gender and limb groups. 

Data from ethnic-specific subsamples of young (18–39 years) subjects with normal BMI (18.5–24.9 kg/m^2^) were adopted to establish cutoff values for the investigated variables using 1 or 2 standard deviations below (for the lean and muscle mass amount) or above (for the lean distribution) each mean. We reported the rounded figures for all cutoff values, as suggested by the EWGSOP-2 consensus [2], to facilitate their use in the clinical practice. 

The threshold for statistical significance was set to *p* = 0.05. Statistical tests were performed using the SPSS v. 20 (IBM Corporation, Armonk, NY, USA) software package.

## 3. Results

A total sample of 10,014 participants (53.8% males and 46.2% females) was analyzed: anthropometric variables and DXA-derived lean mass indices of the sample according to gender and ethnicity are reported in Table 1.

Figure 1 shows the results of the different TBSMM estimations obtained for a representative Caucasian male subject (aged 44 years, ALM 25.6 kg) by using the six equations: TBSMM values ranged from 28.2 kg to 29.6 kg, and the lowest value was obtained with Equation #6.

Similar to this example, the analysis of the group data showed TBSMM values that ranged from 28.2 kg to 32.0 kg in males and from 17.7 kg to 21.6 kg in females (Table 2).

Significant differences (*p* < 0.0001 for all comparisons) were found among the six TBSMM estimates obtained for each of the four groups (Table 2, last row). Post hoc analyses showed that the TBSMM estimates obtained with Equation #6 were significantly (*p* < 0.0001 for all comparisons) lower for males of the two ethnic groups and for African American females in comparison with the estimates obtained with the other five equations. In Caucasian females, the TBSMM estimates obtained with Equation #6 were significantly (*p* < 0.0001 for all comparisons) lower in comparison with the estimates obtained with Equations #1, #2, and #3. 

Results reported in the following were obtained with Equation #6.

**Figure 2:**
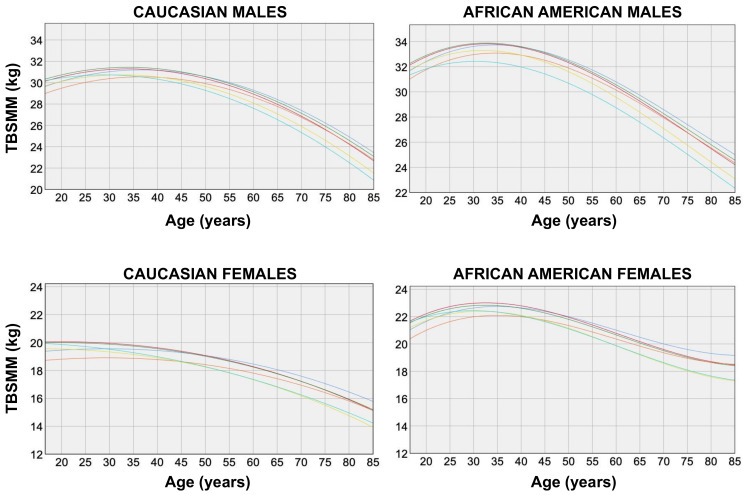
Total body skeletal muscle mass (TBSMM) vs. age in males (top panels) and females (bottom panels) of the two ethnic groups. TBSMM estimations were obtained according to the six equations previously proposed by Kim et al. [10,11]: blue, Equation #1; red, Equation #2; green, Equation #3; orange, Equation #4; yellow, Equation #5; light blue, Equation #6.

Figure 2 shows the variation of TBSMM estimates obtained with the six equations according to age and ethnicity in males and females.

For both genders of the two ethnic groups, TBSMM estimates remained almost stable or increased until the age of 30 years (average peak values obtained with Equation #6 were 31 kg and 32 kg in Caucasian and African American males, respectively, who reached their peaks at 30 years of age; and average peak values of 20 kg and 22 kg were found in Caucasian and African American females, respectively, who reached their peaks at 18 and 30 years of age, respectively) and decreased after this age (compared with the average peak values, the mean values at the age of 65 years decreased by 4 kg and 5 kg in Caucasian and African American males, respectively, and decreased by 2 kg and 3 kg in Caucasian and African American females, respectively). 

The rates of change of the TBSMM estimated from the slope of the regression lines fitting the data (obtained with Equation #6) from the peak to the lowest values were −0.18 kg/year and −0.17 kg/year for Caucasian and African American males, respectively (i.e., the muscle loss was 6% per decade and 5% per decade for Caucasian and African American males, respectively) and −0.10 kg/year for both Caucasian and African American females (i.e., the muscle loss was 5% per decade and 4.5% per decade for Caucasian and African American females, respectively).

**Figure 3:**
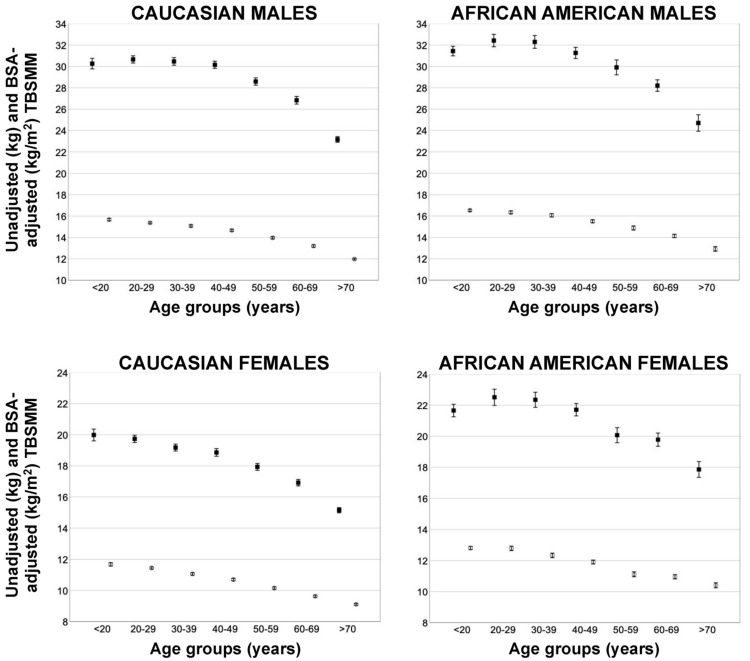
Unadjusted (black squares) and body surface area (BSA)-adjusted (white circles) mean values (obtained with Equation #6) of total body skeletal muscle mass (TBSMM) according to the age group in males (top panels) and females (bottom panels) of the two ethnic groups. Error bars indicate 95% confidence interval.

Figure 3 shows the unadjusted and BSA-adjusted TBSMM mean values (obtained with Equation #6) according to the age group. 

Unadjusted TBSMM values remained almost stable or increased during the second, third, and fourth decades (until the age of 40 years) and decreased progressively during the subsequent decades. Adjusting TBSMM for BSA resulted in different trends; in fact, the BSA-adjusted TBSMM values peaked in the second decade and decreased progressively during the subsequent decades for both genders of the two ethnic groups.

**Figure 4:**
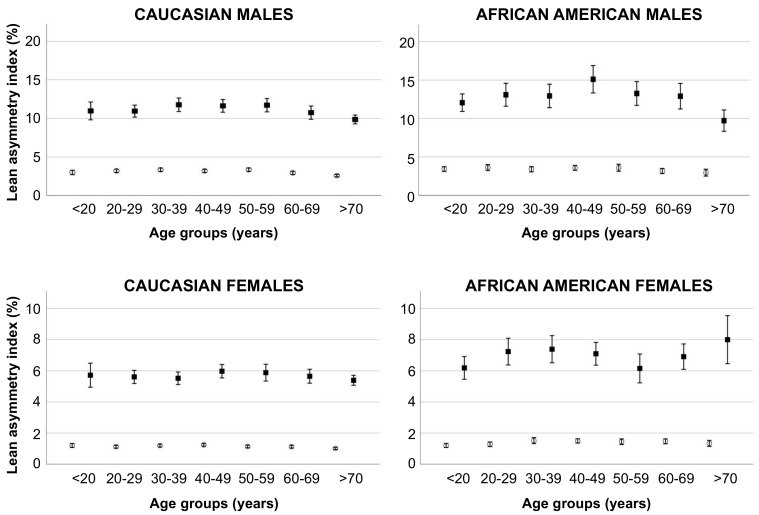
Mean values of the lean asymmetry index for the upper limb (white circles) and lower limb (black squares) according to the age group in males (top panels) and females (bottom panels) of the two ethnic groups. Error bars indicate 95% confidence interval.

The lean asymmetry indices obtained for the upper and lower limbs in the representative male subject reported in Figure 1 were 4.3% for the upper limbs and 5.4% for the lower limbs. As shown in Figure 4, these values were similar for the upper limbs and lower for the lower limbs with respect to those obtained in the whole group of Caucasian males of the corresponding age group (40–49 years). Figure 4 also shows that the lean asymmetry indices were significantly (*p* < 0.0001 for all analyses) higher for the lower limbs compared with the upper limbs for both genders of the two ethnic groups and were also higher for males of the two ethnic groups compared with females.

A subsample of 1915 young (aged 18–39 years) participants (52.9% males and 47.1% females) with a normal BMI (18.5–24.9 kg/m^2^) was defined as the reference population. Their mean and standard deviation values for all DXA-derived indices of lean/muscle mass amount and lean distribution are reported in Table 3. The cutoff values for all DXA-derived indices were generated using 1 or 2 standard deviations below (for the indices of lean/muscle mass amount) or above (for the lean distribution index) each mean to define, respectively, moderately or severely low/asymmetric values of lean/muscle mass amount and lean distribution.

## 4. Discussion

This study investigated the age-related TBSMM changes and the between-limb asymmetry in upper- and lower-extremity lean mass in a large sample of subjects of both genders of different ethnic groups. The main findings of our study were: (i) TBSMM estimates obtained with Equation #6 were lower in comparison with the estimates obtained with the other five equations (i.e., the different equations are not interchangeable), (ii) the age-related decline of TBSMM absolute values (i.e., age-related muscle loss) was between 5% and 6% per decade in males and between 4.5% and 5.0% per decade in females, (iii) the adjustment of TBSMM for BSA showed that muscle mass peaked in the second decade and decreased progressively during the subsequent decades for both genders of the two ethnic groups, and (iv) the lean asymmetry indices were higher for the lower limbs compared with the upper limbs and were higher for males compared with females. 

The different equations developed and validated by Kim et al. [10,11] to predict TBSMM from DXA-derived ALM and anthropometric variables do not seem to be interchangeable to us. Using whole-body magnetic resonance imaging (MRI) as the reference, Kim et al. [10] developed and validated the first three equations (#1, #2, and #3) to estimate TBSMM with the inclusion of the inter- and intra-muscular adipose tissue (IMAT: the adipose tissue found between and within muscles) as part of the TBSMM. However, the presence of IMAT within the muscle compartment may bias the muscle mass estimates when subjects are studied across different gender, age, weight, ethnic, and disease groups. The last three equations (#4, #5, and #6) were developed by Kim et al. [11] for IMAT-free TBSMM prediction. Although the IMAT component is relatively small, especially in healthy subjects, its exclusion (i.e., the correction of the TBSMM overestimation) may explain the differences observed between the two sets of equations. In fact, the TBSMM estimates obtained with the last three equations were lower (difference of ~1 kg) in comparison with the estimates obtained with the first three equations. An additional explanation for the different results obtained with different equations is represented by the differences in the considered variables. Two equations (#1 and #4) adopt the ALM as the only predictor variable for TBSMM, while two equations (#2 and #5) adopt ALM and age, one equation (#3) adopts ALM, age, and gender, and one equation (#6) adopts ALM, age, gender, ethnicity and also the interactions between different variables. On the basis of the differences between the properties and results of the various equations, Kim et al. [11] suggested that Equation #5 should prove practical to apply in the clinical setting given its simple model. However, given that Equation #6 takes into account more variables and also considers their interactions, we suggest using this model for TBSMM estimation that should be systematically included in DXA reports because of its relevance for different disciplines (nutrition, endocrinology, sports medicine, and rehabilitative medicine).

The age-related decrease in TBSMM absolute values we observed in the present study is in agreement with previous observations showing a curvilinear relationship between age and ALMI [15] and between age and MRI-derived muscle mass [16], with a change in the slope of the regression line occurring at ~45 years (for both men and women) and with a decrease in muscle mass that approximated 1.9 and 1.1 kg/decade in men and women, respectively.

The age-related decrease of the BSA-adjusted TBSMM values started after the second decade for both males and females of the two ethnic groups; this observation is consistent with previous data showing that the increase in body weight observed between 20 and 40 years of age is not associated with a corresponding increase in muscle mass since the composition of weight gain before age 40 years is predominantly fat [16]. Consequently, muscle mass adjusted by body weight (or another weight-related parameter such as height or BSA) is reduced relatively early in life [16].

Normalization of lean and muscle mass for body size is a standard approach in clinical evaluation and recommended by the guidelines to enable the comparisons among different subjects independently of their body size. However, no agreement exists about the best body size variable and scaling methodology for lean and muscle mass normalization. Skeletal muscle mass scales to height with power of ~2 [17]; therefore, scaling lean mass to height with a power of 2 became the most common approach for sarcopenia assessment in both research and clinical practice. However, it has been previously observed that the use of muscle mass normalized to height-squared may underestimate the prevalence of low muscle mass, particularly among overweight and obese patients [18,19]. A very recent study by Brown et al. [20] also found that scaling skeletal muscle area (obtained by a single-slice abdominal computed tomography image acquired at the level of the third lumbar vertebra) to height with a power of 2 to identify patients with sarcopenia may result in misclassification. Moreover, it has been observed that muscle mass normalized to BSA was closely associated with muscle strength and physical performance [19]. BSA estimation is already widely adopted in different clinical settings for modeling water loss, in particular for burn injuries, and calculating chemotherapeutic medication dosages [21]. This estimation can easily be obtained through a simple anthropometry-based equation such as the Du Bois formula [14]. Although this formula is the most commonly used, other equations are available, and discrepancies have been documented between different BSA estimation equations [22]. Future studies are required to identify (and standardize the use of) the best equation for BSA estimation that can be adopted for normalization of muscle mass. Alternatively, BSA could be assessed through a digital anthropometric approach [21,23]. In fact, availability and diffusion of new devices such as three-dimensional optical body scanners will make the BSA estimation accurate, easily feasible, and therefore available to be systematically incorporated in the anthropometric assessment of patients before or after DXA.

A vast number of daily physical activities and sports activities involve bilateral limb movement, especially for the lower extremity (e.g., chair standing, walking, stair climbing, running), and the ability to perform these activities can therefore be affected by bilateral limb muscle function that can, in turn, be affected (at least partly) by the between-limb symmetry in lean mass. Consistently, previous studies performed in athletes have shown that leg lean mass asymmetry affects kicking performance [24], jumping performance [25], and motor performance during multi-joint closed-kinetic-chain movements [26]. Lee et al. [8] have found a significant negative relationship between the asymmetry in lower-extremity lean mass and gait speed in community-dwelling older adults. We are not aware of previous studies investigating the between-limb asymmetry in upper- and lower-extremity lean mass in a large group of subjects of different genders and ethnic groups. We found that the between-limb asymmetry of lean mass was higher for the lower limbs compared with the upper limbs and showed a gender difference. Moreover, on the basis of data obtained in the subsamples of young subjects we identified the following thresholds and ranges to distinguish between different categories of asymmetry of the lean mass distribution: (i) low (i.e., physiological) asymmetry: upper-limb AI in females 1–3%; upper-limb AI in males 3–7%; lower-limb AI in females 5–13%; lower-limb AI in males 10–25%; (ii) high (i.e., pathological) asymmetry: upper-limb AI in females >3%; upper-limb AI in males >7%; lower-limb AI in females >13%; and lower-limb AI in males >25%.

From a clinical perspective, the observed inter-limb and inter-gender variability of the lean mass distribution highlights the appropriateness of using strength and conditioning interventions as a strategy to maintain or increase limb symmetry, especially for the lower limbs, in an effort to optimize performance while minimizing injury incidence, decreased ability to perform the daily life activities, and disability. 

From a methodological perspective, the observed lean mass asymmetries also have relevant implications for the preparation of DXA reports. For body composition assessment, it is desirable to scan the entire body. However, vertical and horizontal offset scanning techniques were also devised with matching analysis techniques to piece together complete whole-body scans from partial scans. For very wide patients (i.e., obese patients), the subject can be offset horizontally such that in one scan, all of the body can be estimated even though one of the arms, legs, or both cannot be acquired. This scanning method is called offset scanning in general (reflection mode on Hologic systems and mirror mode on GE systems) [4,27]. Although it has been demonstrated that half-body scanning can accurately predict whole-body composition [28,29], it could be suggested that high values of between-limb asymmetry in lean (and fat) mass may bias the composition estimation for the partially scanned limb from the fully scanned limb. 

Different cut-off points for ALM and ALMI have been proposed to discriminate between normal and low lean mass [6,7]. The most commonly adopted cut-off points were those proposed by the EWGSOP-2 consensus (ALMI < 7.0 kg/m^2^ for men and <5.5 kg/m^2^ for women) [2], which are similar to the ALMI cut-points identified (as values of 2 standard deviations below the means of the two gender and ethnic groups) in the present study (Table 3). However, it has previously been observed that the prevalence of sarcopenia is highly dependent on the applied diagnostic criteria [30,31,32]. Moreover, the use of ALMI to identify patients with sarcopenia may result in misclassification [18,19]. The new sarcopenia index (TB-MAXI) we have proposed could overcome some limitations of the previously proposed indices because it considers TBSMM (and not only its appendicular proxy) and because the normalization factor is an anthropometrical variable (BSA) that is related to both height and weight. On the basis of the data obtained in the subsamples of young subjects we identified the following thresholds to distinguish between low and normal TB-MAXI: (i) 10.0 kg/m^2^ and 11.0 kg/m^2^ in Caucasian and African American females; and (ii) 12.5 kg/m^2^ and 14.5 kg/m^2^ in Caucasian and African American males.

## 5. Conclusions

The present study proposes TB-MAXI and lean AI that can be used (and included in DXA reports) as clinically relevant markers for muscle amount and lean distribution. The BSA adjustment could provide an appropriate normalization of the muscle compartment, especially in patients misclassified by commonly used sarcopenia indices. Further studies are required to establish the most accurate equation or approach to estimate the BSA and to analyze the functional relevance of the proposed TB-MAXI and lean AI cut-off points (i.e., their association with functional or adverse outcomes) in sarcopenic patients.

## Figures and Tables

**Table 1:** Anthropometric variables and DXA-derived lean mass indices of the sample (*n* = 10,014) according to gender and ethnicity. Data are reported as means ± standard deviations. ALM: appendicular lean mass; ALMI: appendicular lean mass index; BMI: body mass index.

Variable	Caucasian Males (*n* = 3770)	African American Males (*n* = 1619)	Caucasian Females (*n* = 3328)	African American Females (*n* = 1297)
Age (years)	49.4 ± 19.9	41.5 ± 18.7	50.3 ± 20.3	42.6 ± 18.8
Weight (kg)	83.4 ± 14.0	80.8 ± 14.9	67.9 ± 12.5	72.1 ± 13.5
Height (m)	1.77 ± 0.07	1.77 ± 0.07	1.63 ± 0.07	1.63 ± 0.07
BMI (kg/m^2^)	26.7 ± 3.9	25.9 ± 4.3	25.7 ± 4.4	27.1 ± 4.5
ALM (kg)	25.5 ± 4.1	27.7 ± 4.7	16.4 ± 2.7	19.0 ± 3.2
ALMI (kg/m^2^)	8.14 ± 1.06	8.84 ± 1.23	6.20 ± 0.85	7.14 ± 1.00

**Table 2:** Mean ± standard deviation values of the total body skeletal muscle mass (TBSMM) estimates obtained for the four groups by using the six equations proposed by Kim et al. [10,11].

TBSMM (kg)	Caucasian Males (*n* = 3770)	African American Males (*n* = 1619)	Caucasian Females (*n* = 3328)	African American Females (*n* = 1297)
Equation #1	29.3 ± 4.9	31.9 ± 5.5	18.6 ± 3.3	21.6 ± 3.8
Equation #2	29.2 ± 5.0	31.9 ± 5.5	18.6 ± 3.4	21.7 ± 3.8
Equation #3	29.4 ± 4.8	32.0 ± 5.4	18.5 ± 3.2	21.6 ± 3.7
Equation #4	28.6 ± 4.9	31.3 ± 5.5	17.9 ± 3.3	21.0 ± 3.8
Equation #5	28.4 ± 5.1	31.3 ± 5.6	17.7 ± 3.5	21.0 ± 3.9
Equation #6	28.2 ± 4.9	30.5 ± 4.9	17.9 ± 3.0	21.1 ± 3.5
*p* value	<0.0001	<0.0001	<0.0001	<0.0001

**Table 3:** Mean and standard deviation values for all DXA-derived indices of lean/muscle mass amount and distribution obtained in the reference population of 1915 young (aged 18–39 years) participants with normal body mass index (18.5–34.9 kg/m^2^). TLM: total lean mass; LMI: lean mass index (total lean mass/height^2^); ALM: appendicular lean mass; ALMI: appendicular lean mass index (appendicular lean mass/height^2^); TBSMM: total body skeletal muscle mass (obtained with Equation #6 by Kim et al. [11]); TB-MAXI: total body muscularity assessment index (total body skeletal muscle mass/body surface area (obtained with equation by Du Bois and Du Bois [14]); AI: asymmetry index.

	**Caucasian Males** **(*n* = 603)**			**African American Males** **(*n* = 410)**		
	**Mean ± SD**	**−1 SD**	**−2 SD**	**Mean ± SD**	**−1 SD**	**−2 SD**
TLM (kg)	54.1 ± 5.5	48.6	43.1	55.1 ± 6.3	48.8	42.5
LMI (kg/m^2^)	16.1 ± 0.5	15.6	15.1	16.4 ± 0.1	16.3	16.2
ALM (kg)	24.4 ± 2.9	21	19	26.5 ± 3.5	23	19
ALMI (kg/m^2^)	7.71 ± 0.67	7.0	6.5	8.37 ± 0.80	7.5	7.0
TBSMM (kg)	27.9 ± 3.5	24	21	30.2 ± 3.1	27	24
TB-MAXI (kg/m^2^)	14.80 ± 1.18	13.5	12.5	16.20 ± 0.92	15.0	14.5
	**Mean ± SD**	**+1 SD**	**+2 SD**	**Mean ± SD**	**+1 SD**	**+2 SD**
Upper Limb AI (%)	2.9 ± 2.1	5.0	7.1	3.1 ± 2.5	5.6	8.1
Lower Limb AI (%)	9.7 ± 7.9	17.6	25.5	10.3 ± 8.5	18.8	27.3
	**Caucasian Females (*n* = 641)**			**African American Females (*n* = 261)**		
	**Mean ± SD**	**−1 SD**	**−2 SD**	**Mean ± SD**	**−1 SD**	**−2 SD**
TLM (kg)	38.3 ± 3.8	34.5	30.7	38.6 ± 4.1	34.5	30.4
LMI (kg/m^2^)	13.1 ± 0.04	13.1	13.0	13.5 ± 0.1	13.4	13.3
ALM (kg)	16.1 ± 2.0	14	12	17.3 ± 2.2	15	13
ALMI (kg/m^2^)	5.95 ± 0.57	5.5	5.0	6.51 ± 0.63	6.0	5.0
TBSMM (kg)	18.6 ± 1.8	17	15	20.2 ± 1.9	18	16
TB-MAXI (kg/m^2^)	11.25 ± 0.76	10.5	10.0	12.47 ± 0.80	12.0	11.0
	**Mean ± SD**	**+1 SD**	**+2 SD**	**Mean ± SD**	**+1 SD**	**+2 SD**
Upper Limb AI (%)	1.1 ± 0.8	1.9	2.7	1.1 ± 0.8	1.9	2.7
Lower Limb AI (%)	5.0 ± 4.0	9.0	13.0	5.8 ± 4.8	10.6	15.4

## Data Availability

The data that support the findings of this study are available from the corresponding author upon reasonable request.
